# Preventive effect of irsogladine or omeprazole on non-steroidal anti-inflammatory drug-induced esophagitis, peptic ulcers, and small intestinal lesions in humans, a prospective randomized controlled study

**DOI:** 10.1186/1471-230X-13-85

**Published:** 2013-05-14

**Authors:** Takanori Kuramoto, Eiji Umegaki, Sadaharu Nouda, Ken Narabayashi, Yuichi Kojima, Yukiko Yoda, Kumi Ishida, Ken Kawakami, Yosuke Abe, Toshihisa Takeuchi, Takuya Inoue, Mitsuyuki Murano, Satoshi Tokioka, Kazuhide Higuchi

**Affiliations:** 1 2nd Department of Internal Medicine, Osaka Medical College, 2-7 Daigaku-machi, Takatsuki, Osaka, Japan

**Keywords:** Small-intestinal injury, NSAIDs, Irsogladine, Omeprazole

## Abstract

**Background:**

Proton-pump inhibitors such as omeprazole are a standard treatment to prevent non-steroidal anti-inflammatory drug-induced upper gastrointestinal mucosal injuries. However, it is unclear which drugs may protect against all NSAID-induced digestive-tract injuries. Here, we compare the efficacy of the gastromucoprotective drug irsogladine with omeprazole in preventing NSAID-induced esophagitis, peptic ulcers, and small-intestinal mucosal injury in healthy subjects.

**Methods:**

Thirty-two healthy volunteers were assigned to an irsogladine group (Group I; n = 16) receiving diclofenac sodium 75 mg and irsogladine 4 mg daily for 14 days, or an omeprazole group (Group O; n = 16) receiving diclofenac sodium 75 mg and omeprazole 10 mg daily for 14 days. Esophagitis and peptic ulcers were evaluated by esophagogastroduodenoscopy and small-intestinal injuries by capsule endoscopy, fecal calprotectin, and fecal occult blood before and after treatment.

**Results:**

There was no significant difference between Group I and Group O with respect to the change in lesion score in the esophagus, stomach, and duodenum before and after treatment.NSAID treatment significantly increased the number of small intestinal mucosal breaks per subject by capsule endoscopic evaluation, from a basal level of 0.1 ± 0.3 up to 1.9 ± 2.0 lesions in Group O (p = 0.0002). In contrast, there were no significant changes in the mean number of mucosal breaks before and after co-treatment in Group I (0.3 ± 0.8 to 0.5 ± 0.7, p = 0.62), and the between-group difference was significant (p = 0.0040). Fecal calprotectin concentration, when the concentration before treatment was defined as 1, was significantly increased both in Group O (from 1.0 ± 0.0 to 18.1 ± 37.1, p = 0.0002) and Group I (from 1.0 ± 0.0 to 6.0 ± 11.1, p = 0.0280); the degree of increase in Group O was significantly higher compared with that in Group I (p<0.05). In addition, fecal occult blood levels increased significantly in Group O (p = 0.0018), but there was no change in Group I (p = 1.0), and the between-group difference was significant (p = 0.0031).

**Conclusion:**

Irsogladine protected against NSAID-induced mucosal injuries throughout the gastrointestinal tract, from esophagus to small intestine, significantly better than omeprazole.

**Trial registration:**

This study was registered in the UMIN Clinical Trials Registry (Registry ID number; UMIN000008114)

## Background

Gastroduodenal mucosal lesions are a well-known adverse effect of non-steroidal anti-inflammatory drugs (NSAIDs) [1]. Recently, the serious problem of NSAID-induced small-intestinal damage has become a topic of great interest to gastroenterologists since capsule endoscopy and balloon enteroscopy have become available for the detection of small-intestinal lesions [2]. Recent studies have shown that 55–68% of patients taking NSAIDs have some mucosal damage in the small intestine [3-5]. Such lesions are of great concern in clinical settings, and methods for their treatment and prevention must be devised as soon as possible. Proton-pump inhibitors (PPIs) are a standard treatment for the prevention of NSAID-induced upper gastrointestinal mucosal injuries. However, it is not clear whether PPIs are effective in the lower digestive tract, where there is no acid. Irsogladine (2,4-diamino-6-[2,5-dichlorophenyl]-*s*-triazine), a drug for the treatment of gastric ulcers that is widely used in Japan, Korea and China, protects the gastric mucosa by enhancing the mucosal integrity of the stomach through the facilitation of gap-junctional intercellular communication [6]. Irsogladine also prevents the development of intestinal lesions induced by indomethacin in rats [7]. Irsogladine can be expected to be effective not only in the stomach but also in other parts of the digestive tract. Previous studies on the prevention of NSAID-induced digestive tract injuries by various drugs [8-10] have been limited to the upper digestive tract or the small intestine individually, and there have been no studies of the entire digestive tract from the esophagus through the stomach, duodenum, and small intestine. It would be of great benefit if a single drug could be used to manage NSAID-induced injuries of both the upper and lower digestive tract. In the present study, we compared the efficacy of irsogladine and omeprazole in preventing NSAIDs-induced esophagitis, peptic ulcers, and small-intestinal mucosal injury in healthy subjects by using multidimensional assessment; that is, esophagastroduodenoscopic evaluation, capsule endoscopic evaluation, fecal calprotectin concentration and occult fecal blood test.

## Methods

### Subjects

The study of 32 healthy volunteers was conducted prospectively from April to August 2010 at Osaka Medical College Hospital. Subjects eligible for inclusion were healthy adults who 1) were aged between 20 and 79 years of age at the time of obtaining consent, 2) had freely given their fully informed consent based on their full understanding, and 3) had taken no medication during the one-month period before the start of the study. The exclusion criteria were 1) a history of peptic ulcer or gastrointestinal bleeding, 2) significant hepatic, renal, heart, or respiratory disease, 3) a history of gastrointestinal surgery other than appendectomy, 4) oral use or planned oral use of a drug other than an antiulcer drug, 5) alcohol or chemical dependency, 6) a history of intestinal obstruction or suspected gastrointestinal obstruction on other tests, 7) a lack of consent to the surgery required if the capsule endoscope was retained in the body, and 8) a determination by the investigator, at his discretion, that a subject was ineligible for participation in the study for any other reason. All subjects received oral and written explanation of the study prior to participation and gave written informed consent. The study was conducted in accordance with the Declaration of Helsinki (1995) after the protocol had been approved by the Ethics Review Committee of Osaka Medical College.

### Protocol

This was a prospective, randomized, study. Every day for two weeks, the irsogladine group (Group I) received diclofenac sodium 75 mg plus irsogladine maleate 4 mg, and the omeprazole group (Group O) received diclofenac sodium 75 mg plus omeprazole 10 mg. The dose of diclofenac sodium was determined based on the dose approved by the Japanese Ministry of Health and Welfare and the doses used in other clinical trials [8-10]. Generally, the dosage of a PPI used for the prevention of NSAID-induced gastric ulcers is half the dosage used for the treatment of gastric ulcers in Japan. On this basis, we determined that the appropriate dosage of omeprazole should be 10 mg/day.

The subjects were assigned to either Group I or Group O prior to the study. Bowel preparation, capsule endoscopy with a PillCam™SB video capsule (Given Imaging, Yoqneam, Israel) and image evaluation were conducted as previously reported [11]. We conducted a preliminary analysis of the results of these baseline capsule endoscopy examinations to determine subject eligibility for the remainder of the study. Images were analyzed with the software program Rapid Reader 4 (Given Imaging). Lesions were evaluated according to the Los Angeles classification or the Lanza score [12] by esophagogastroduodenoscopy, and the number of small-intestinal mucosal lesions was assessed by capsule endoscopy, serum biochemistry, fecal occult blood, and fecal calprotectin before and after two weeks of treatment. A diagnosis of *Helicobacter pylori* (*H. pylori*) infection was confirmed by a blood antibody test at the beginning of the trial.

### Esophagogastroduodenoscopy

To standardize the reporting criteria for the endoscopic findings, the two endoscopists (T. K. and E.U.) attended each other’s endoscopic sessions before and regularly during the trial.

### Capsule endoscopy

**Figure 1:**
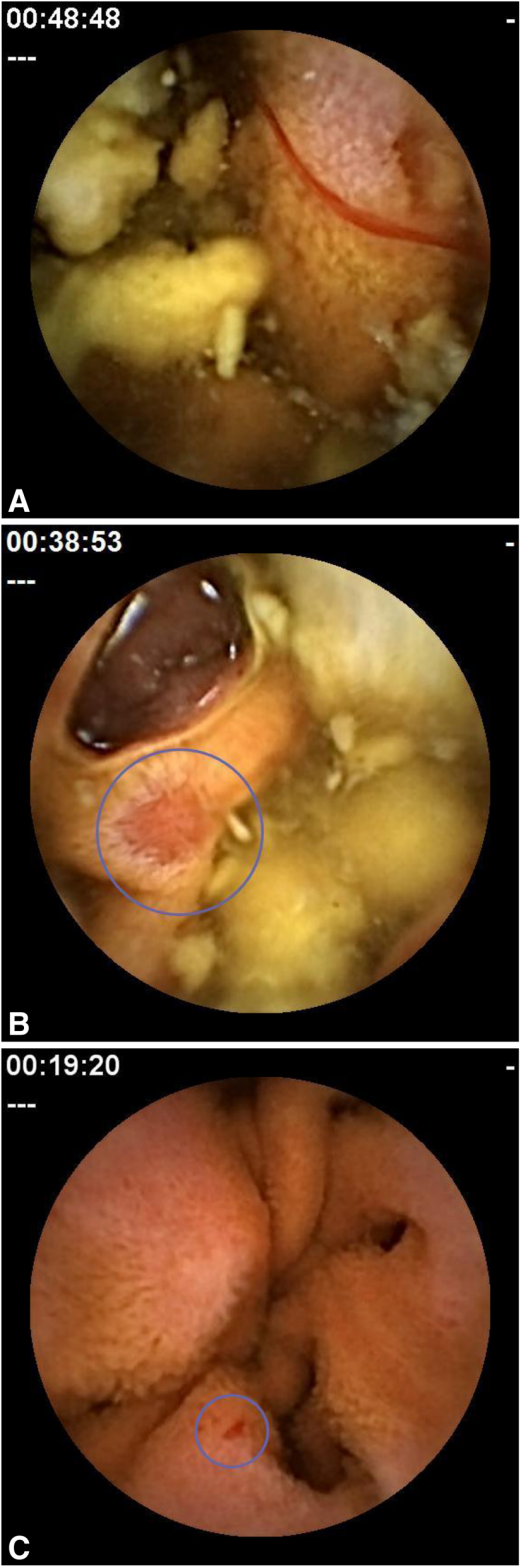
Example photographs by capsule endoscopy (A) typical bleeding, (B) mucosal break, (C) reddish lesions.

Mucosal breaks in the small intestine were defined as lesions with slough surrounded by erythema, corresponding to the grade 2 category of Goldstein et al. [4]. Typical examples of the bleeding, mucosal breaks and reddish lesions found in this study are shown in Figure 1A–C. Reddish lesions, such as reddened folds, denuded areas, and petechiae, were grouped in a single classification: reddened lesions. Mucosal breaks, reddened lesions and bleeding were identified and evaluated by independent blinded reviewers as described below. The number of mucosal breaks, reddened lesions and sites of bleeding in the small intestine found at baseline and post-treatment by capsule endoscopy was calculated for each subject and compared between Groups I and O. The percentage of subjects with at least one mucosal break in each treatment group was also calculated.

Investigators who were to evaluate the results of capsule endoscopy of the small intestine were required to attend a standardized training session on the use of the Given Diagnostic System. These two investigators (T.K. and E.U.) independently assessed the capsule endoscopic images under blinded conditions. Positive findings were classified as either mucosal bleeding or mucosal injury. If the two observers recorded different findings, they discussed the case until they reached agreement.

### Noninvasive tests of intestinal damage

Subjects collected a stool sample for determination of fecal calprotectin as a measure of intestinal inflammation at baseline and the final visit. Stools were frozen within 12 h of receipt and stored at −20°C for subsequent analysis with an enzyme-linked immunosorbent assay kit (Immundiagnostik, Bensheim, Germany) as previously described [13]. Results are expressed as micrograms of calprotectin per gram of stool, and a cutoff value of 50 μg/g stool was used, as recommended by the manufacturer [14]. The fecal calprotectin value suffers the problems of variation, so we determined to use the fold increase after treatment when the calprotectin concentration before treatment was set to 1. Before and after the study, fecal occult blood was assessed with the tetramethylbenzidine and guaiac tests by using occult fecal blood slide kits from Shionogi Pharma (Osaka, Japan). In both tests, the color intensity of the oxidation product was assigned to one of three categories, +, ± or −, and on this basis differences between before and after treatment were denoted “exacerbation”, “invariable” or “improvement”. The hemoglobin and transferrin antibody tests for occult fecal blood were performed with an OC-Micro analyzer (Eiken, Tokyo, Japan). Generally, fecal occult blood is influenced by the intake of meat, fish, bright red, green or yellow vegetables, and so on. Therefore, we explained to our subjects how these foods affect the results of the occult blood tests(the tetramethylbenzidine test and the guaiac test), and suggested that they pay attention to their food intake during the period 4 days prior to the examination date.

### Sample size

The sample size was based on our estimation of the proportion of subjects that would be expected to exhibit mucosal breaks at post-treatment by capsule endoscopy. We estimated that the incidence of mucosal injuries would be approximately 20% in the irsogladine group, on the basis of a preliminary study by Niwa et al. [8] showing that the incidence of NSAID-induced small-intestinal lesions was lower in subjects on daily rebamipide (20%) than in subjects on placebo (80%). In rats, irsogladine suppresses indomethacin-induced small-intestinal lesions as effectively as rebamipide [7]. In addition, we estimated that the incidence of mucosal injuries would be approximately 70% in the control group, because a recent study found small-intestinal lesions in 55–68% of subjects taking NSAIDs [3,4]. Thus, 15 subjects would need to be recruited to each group (30 subjects in total) for a chi-square test, a significance level of 5% (two-sided), a power of 80%, and equal allocation. On the assumption that two subjects would not be able to complete the study, a minimum of 32 subjects was required.

### Randomization

A coordinator performed a simple fixed-allocation randomization by using a block-randomization scheme. Random numbers were generated by SAS (SAS Institute, Cary, NC, USA).

### Statistics

For continuous or categorical variables, the statistical significance of differences between groups was determined with the t test or Wilcoxon rank-sum test, and the statistical significance of differences within a group was determined with the Wilcoxon signed-rank test. For binary variables, the statistical significance of differences between groups was determined with the chi-square test. All reported p values are two-sided, and values of less than 0.05 were considered to indicate statistically significant differences. All statistical values were calculated with SAS Ver. 9.2 (SPSS, Chicago, IL, USA), Windows Edition.

## Results and discussion

### Analysis of subjects

The 32 subjects were randomly assigned to either Group I or Group O and underwent baseline esophagogastroduodenoscopy and capsule endoscopy. None of the subjects had significant findings in the esophagus through to the small intestine, and all 32 were considered eligible for the study. The characteristics of each group’s subjects, including age, sex, *H. pylori* infection status, fecal hemoglobin concentration, and the numbers of mucosal breaks, reddened lesions and sites of bleeding, are shown in Table 1.

**Table 1:** Characteristics of subjects at baseline that underwent full analysis

	**Irsogladine group**	**Omeprazole group**	**p value**
No. of subjects	16	16	
Age (years) (mean ± SD)	25±4	25±4	NS
Sex (M/F)	10/6	11/5	NS
*H. pylori* infection status (+/−)	1/15	1/15	NS
Fecal hemoglobin concentration (mg/dL) (mean ± SD)	14.1 ± 2.0	14.4 ± 1.2	NS
Number of mucosal breaks (mean ± SD)	0.3 ± 0.8	0.1 ± 0.3	NS
Number of reddened lesions (mean ± SD)	0.2 ± 0.4	0.6 ± 0.8	NS
Number of sites of bleeding (mean ± SD)	0.0 ± 0.0	0.1 ± 0.3	NS

NS = not significant.

### Esophagogastroduodenoscopy

By the Los Angeles classification, no esophageal mucosal injuries were observed in either group before or after treatment. In both groups, all 16 subjects were grade O(no mucosal breaks) both before and after treatment(Table 2). There was no significant difference between Group O (from 0.6 ± 0.9 to 1.5 ± 1.1) and Group I (from 0.5 ± 1.1 to 0.9 ± 1.0) in the gastric Lanza score either before or after treatment (p = 0.20). A similar result was obtained for the duodenal Lanza scores (Group O, 0.0 ± 0.0 to 0.4 ± 0.8; Group I, 0.0 ± 0.0 to 0.4 ± 0.9; p = 0.94) (Table 2).

**Table 2:** The Los Angeles classification and Lanza scores at baseline and after treatment

	**Baseline**	**Post-treatment**	**p value**^**1**^
Irsogladine group			
Los Angeles classification	Grade O (16/16)	Grade O (16/16)	
Lanza scores (stomach) (mean ± SD)	0.5 ± 1.1	0.9 ± 1.0	NS
Lanza scores (duodenum) (mean ± SD)	0.0 ± 0.0	0.4 ± 0.9	NS
Omeprazole group			NS
Los Angeles classification	Grade O (16/16)	Grade O (16/16)	
Lanza scores (stomach) (mean ± SD)	0.6 ± 0.9	1.5 ± 1.1	NS
Lanza scores (duodenum) (mean ± SD)	0.0 ± 0.0	0.4 ± 0.8	0.0049

^1^P-values are baseline versus post-treatment within groups.

### Capsule endoscopy

**Figure 2:**
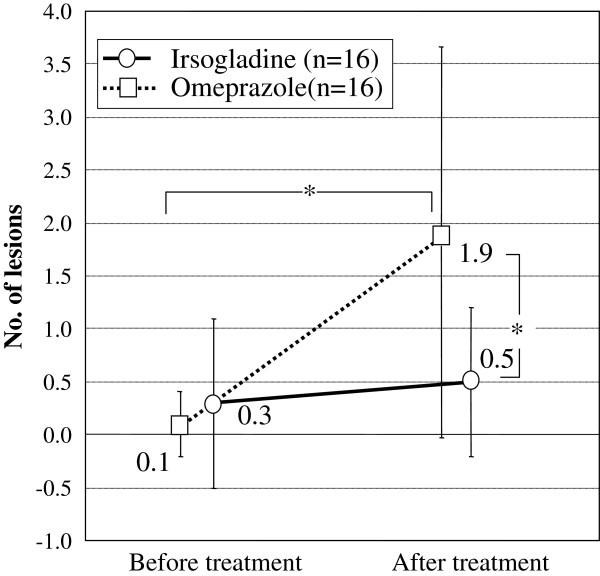
Mean mucosal breaks per subject at post-treatment capsule endoscopy (mean ± SD).

A significantly higher percentage of subjects in Group O (81.3% (13/16)) had mucosal breaks after treatment than in Group I (37.5% (6/16); p = 0.012). The increase in the mean number of small-intestinal mucosal breaks per subject from baseline to study end was significantly greater in Group O (0.1 ± 0.3 to 1.9 ± 2.0, p = 0.0002) than in Group I (0.3 ± 0.8 to 0.5 ± 0.7; , p = 0.62) ( p = 0.0040; Figure 2 and Table 3).; Figure 2 and Table 3). There were no significant differences in the numbers of reddened lesions or sites of bleeding per subject before and after treatment (Table 3).

**Table 3:** Number of small-intestinal lesions per subject by capsule endoscopy at baseline and after treatment

	**Baseline**	**Post-treatment**	**p value**^**1**^
Irsogladine group			
Number of mucosal breaks (mean ± SD)	0.3 ± 0.8	0.5 ± 0.7	NS
Number of reddened lesions (mean ± SD)	0.2 ± 0.4	0.4 ± 0.6	NS
Number of sites of bleeding (mean ± SD)	0.0 ± 0.0	0.2 ± 0.5	NS
Omeprazole group			
Number of mucosal breaks (mean ± SD)	0.1 ± 0.3	1.9 ± 2.0	0.0002
Number of reddened lesions (mean ± SD)	0.6 ± 0.8	1.3 ± 1.7	NS
Number of sites of bleeding (mean ± SD)	0.1 ± 0.3	0.3 ± 0.4	NS

^1^P-values are baseline versus post-treatment within groups.

### Fecal calprotectin

**Figure 3:**
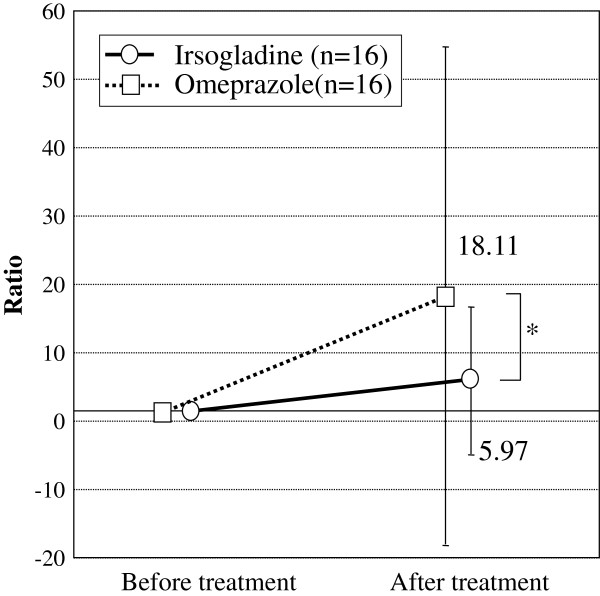
Changes in calprotectin levels after two weeks’ treatment with irsogladine or omeprazole (mean ± SD).

The fecal calprotectin concentration increased after treatment in both groups (Group O: 2400 ± 4000 to 5000 ± 6700, Group I: 14000 ± 35000 to 19000 ± 21000). The median baseline fecal calprotectin concentration increased significantly after treatment in both groups. However, when the calprotectin concentration before treatment was set to 1, the fold increase after treatment was significantly higher in Group O (1.0 ± 0.0 to 18.1 ± 37.1, p = 0.0002) than in Group I (1.0 ± 0.0 to 6.0 ± 11.1, p = 0.028) ( p<0.05, Figure 3).

### Occult blood test of stool

**Figure 4:**
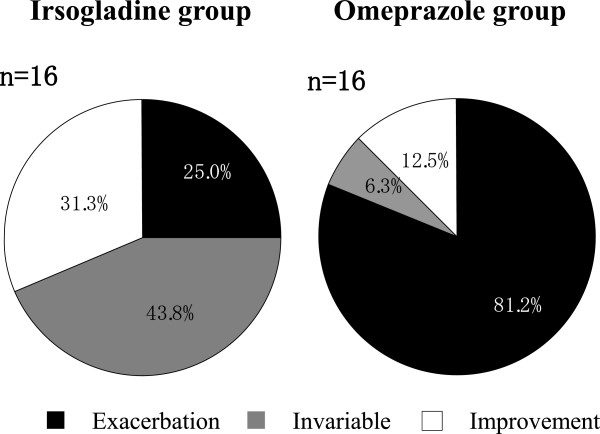
Fecal occult blood after two weeks’ treatment with irsogladine or omeprazole compared to baseline.

As assessed by the tetramethylbenzidine test (Figure 4), fecal occult blood was significantly increased in Group O after treatment compared with before treatment (p = 0.0018), but there was no significant change in Group I(p = 1.0), and there was a significant post-treatment difference between the groups(p = 0.0031). Similar results were obtained with the guaiac test (Group I, exacerbation 25.0% (4/16), invariable 56.3% (9/16), improvement 18.8% (3/16); Group O, exacerbation 81.3% (13/16), invariable 12.5% (2/16), improvement 6.3% (1/16) (p = 0.0031)). By contrast, the fecal occult blood test results obtained by using an antibody to human hemoglobin (Group I, 38.9 ± 13.0 to 35.5 ± 19.5 ng/mL; Group O, 30.8 ± 21.0 to 29.0 ± 24.0 ng/mL) or transferrin (Group I, 13.1 ± 8.0 to 10.9 ± 7.5 ng/mL; Group O, 2.9 ± 5.4 to 3.5 ± 4.7 ng/mL) showed no significant change after treatment compared with before treatment in either group.

### Tolerability

Neither irsogladine nor omeprazole produced any side effects.

### Discussion

Our study demonstrated that short-term administration of irsogladine suppressed NSAID-induced mucosal injuries from the esophagus to the small intestine more effectively than omeprazole. This is the first trial to include a multidimensional assessment of whether a single drug can protect against NSAID-induced lesions in the entire digestive tract from the esophagus to the small intestine.

In previous investigations of the effectiveness of gastroprotective drugs in the prevention of small-intestinal mucosal injuries induced by NSAIDs in volunteers, evaluation was based on capsule endoscopic findings only [8-10], so that the full extent of small-intestinal mucosal injury may not have been appreciated. The use of the biochemical approach (fecal occult blood, calprotectin), in addition to capsule endoscopy, enabled a higher-quality evaluation. In our study, the irsogladine group showed a significantly smaller increase in the number of small-intestinal mucosal injuries by capsule endoscopy, fecal calprotectin, and fecal occult blood compared with the omeprazole group. Irsogladine was originally developed as a drug for the treatment of gastric ulcers and so, as might be expected, we found no significant differences in the esophagus, stomach and duodenum compared with omeprazole. Previous reports suggest that irsogladine exerts various actions, including inhibiting the reduction of gastric mucosal blood flow induced by diclofenac [15], the suppression of free-radical production [16] and the facilitation of gap-junctional intercellular communications [6].

Previous studies have shown that 55–68% of patients taking NSAIDs and omeprazole have some mucosal damage in the small intestine [3,4]. In the present study, the development of lesions, including mucosal breaks, was also not inhibited with omeprazole, with lesions found in 81.3% of subjects in the omeprazole group. In contrast, lesion development was significantly inhibited in the irsogladine group. Prior reports suggest that the activation of gap-junctional intercellular communication by irsogladine leads to a significant decrease in the paracellular permeability of human intestinal epithelial cell monolayers, partly through the up-regulation of claudin-4 [17]. We have found that irsogladine increases mucus secretion and significantly suppresses the decreased mucus response to indomethacin, resulting in the suppression of bacterial invasion as well as the up-regulation of the expression of inducible nitric oxide synthase [7]. The suppression of small-intestinal injuries by irsogladine may be explained partly by the maintenance of intestinal permeability and partly by the stimulation of mucus secretion.

Although misoprostol lowers gastrointestinal complications caused by NSAIDs in addition to preventing endoscopic gastroduodenal ulcers [9,18], it can cause mild diarrhea at a dose of only 600 μg [9]. Therefore, a drug which is safe for use in the prevention of NSAID-induced enteropathy without any adverse gastrointestinal effects is highly desirable. On irsogladine, not only the present study but also a previous study found no adverse drug reactions such as diarrhea or abdominal pain [19].

PPIs are the standard treatment for the prevention of NSAID-induced upper gastrointestinal mucosal injuries; however, this study has shown that the PPI omeprazole was ineffective in the lower digestive tract. Furthermore, Wallace JL et al. reported that PPIs exacerbate NSAID-induced small-intestinal mucosal injuries in experimental animals [20]. A striking effect of PPIs is a significant reduction in the proportion of *Actinobacteria* in the jejunum [20], a finding that strongly suggests that the dysbiosis induced by a PPI is a major contributing factor to the increased susceptibility to NSAID-induced small-intestinal injuries caused by enteric microflora.

The limitation of this study is that we did not include an NSAID monotherapy group, because it would have been ethically unacceptable to administer an NSAID without any prophylactic medicine for gastric ulcer. Therefore, it is unknown whether omeprazole exacerbated small-intestinal lesions. Also, the usefulness of irsogladine is unclear in patients with a history of peptic ulcer or gastrointestinal bleeding when NSAIDs are administered because the study focused on healthy subjects with a low risk of digestive-tract injuries. Additionally, the study was performed in the relatively short period of two weeks, so further study is required to validate the long-term usefulness of irsogladine.

## Conclusions

In conclusion, in healthy volunteers irsogladine did not show significant differences from PPIs in the extent of inhibition of lesion development in the esophagus, stomach, and duodenum, but it did significantly inhibit lesion development in the small intestine compared with PPIs. Therefore irsogladine may be a useful drug in the situation where patients with a low risk of upper digestive tract injuries are administered NSAIDs, to protect the entire digestive tract from the esophagus to the small intestine.

## Abbreviations

PPI: Proton pump inhibitor; NSAID(s): Non-steroidal anti-inflammatory drug(s); H. pylori: Helicobacter pylori.

## Competing interests

The authors have no conflicts of interest to declare.

## Authors’ contributions

Guarantor of the article: TK. Specific author contributions: Principal investigator, subject recruitment, subject evaluation, data collection and manuscript preparation: TK; manuscript preparation and statistical analysis: KH: randomization, subject recruitment, subject evaluation and data collection: TT: subject recruitment, subject evaluation and data collection: EU, SN, K N, YK, YY, KI, KK, YA, TI, MM and ST. All authors read and approved the final manuscript.

## Pre-publication history

The pre-publication history for this paper can be accessed here:

http://www.biomedcentral.com/1471-230X/13/85/prepub
